# Prototype-based sleep micro-structure learning for explainable and robust multimodal recognition of sleep-related conditions

**DOI:** 10.21203/rs.3.rs-9169987/v1

**Published:** 2026-04-01

**Authors:** Guido Gagliardi, Javier García Ciudad, Letizia Micca, Birgitte Rahbek Kornum, Moran Gilat, Antonio Luca Alfeo, Mario G.C.A Cimino, Maarten De Vos

**Affiliations:** 1 Department of Electrical Engineering, KU Leuven, Leuven, Belgium.; 2 Department of Neuroscience, University of Copenhagen, Copenhagen, Denmark.; 3 Department of Rehabilitation Sciences, KU Leuven, Leuven, Belgium.; 4 Dept. of Theoretical and Applied Sciences, eCampus University, Novedrate, Italy.; 5 Department of Information Engineering, University of Pisa, Pisa, Italy.; 6 Department of Development and Regeneration, KU Leuven, Leuven, Belgium.

**Keywords:** Prototype Learning, Explainable AI, Sleep Staging, Physiological Time Series

## Abstract

While sleep is fundamental to human health, sleep disturbances reduce quality of life and constitute risk factors for neurodegenerative diseases including Parkinson’s and Alzheimer’s. Automated sleep staging networks achieve human-level performance on multimodal physiological signals, but they operate as black boxes, limiting clinical trust and preventing the discovery and validation of sleep biomarkers linked to human health status. We propose ProtoSleepNet (PSN), the fist prototype-based sequence-to-sequence sleep staging architecture that achieves human-level sleep staging accuracy while providing interpretability through an intrinsic codebook of learned prototypes. Each prototype captures distinctive sleep microstructure patterns, visualized as physiologically meaningful features across EEG, EOG, and EMG channels. We validate PSN against state-of-the-art approaches on over 10,000 subject recordings across 10 benchmark datasets, demonstrating in-line or superior sleep staging performance, robustness to channel occlusion attacks, and interpretability through a novel explainability framework that translates abstract prototypes into clinically aligned natural-language matching rules. Finally, we show that prototype sequences (prototype-grams) from individual patients encode clinically relevant information: without any disease-specific training, prototype-grams effectively discriminate Parkinson’s and Alzheimer’s disease patients from healthy controls, revealing disease-specific sleep microstructure alterations aligned with known pathophysiology.

## Introduction

Sleep disorders, affecting 10–20% of the population [1], substantially reduce quality of life. Sleep length and quality are closely linked to overall health status, and changes in sleep macro- or micro- architecture can represent early signs of neurodegenerative diseases, such as Parkinson’s disease (PD) and Alzheimer’s dementia (AD) [2]. Additionally, recent evidence identifies sleep disturbances as risk factors for neurodegeneration later in life [3]. Hence, large scale screening for sleep abnormalities could result in earlier diagnosis of sleep related disturbances and, through the promotion of early interventions, improving life quality and reducing the incidence of brain-related disorders.

Sleep assessment involves recording one or multiple nights of polysomnography (PSG) data from a subject and manually labeling each 30-second window in 5 stages (Wake, N1, N2, N3 and REM). Although, this procedure is costly and labor-intensive, screening the wider population could become possible nowadays thanks to available in-home PSG recording hardware [4], or wearable sensors [2], and automatic sleep staging algorithms with accuracies in the range of human inter-rater agreement [5]. These innovations could be suitable for processing of large amounts of sleep data in the broader population.

In the sleep staging context, the most popular algorithms employ Artificial Intelligence (AI) architectures [6–8] to achieve high performances, such as the sequence-to-sequence deep learning framework [9]. This framework consists of an epoch encoder, embedding 30-second windows of data, and a sequence encoder, processing the information flow of L subsequent epochs and transforming them into L sleep stage predictions. This hierarchical structure exploits the longitudinal dependence between sleep states within a sleep cycle to achieve human-level performance outperforming classical epoch-to-epoch sleep staging architectures [10].

The primary goal of these deep learning approaches is to minimize the need for manual sleep scoring in large-scale studies. As discussed earlier, such models already capture the overall sleep macrostructure, i.e., the progression of sleep stages across the night, with performance comparable to human experts. However, two key limitations still hinder their full adoption in clinical practice: first, the opaque decision-making process of such deep learning approaches prevents researchers from fully trusting their predictions; second, while sleep macrostructure forms the basis of conventional sleep scoring, growing interest now focuses on sleep microstructure, describing events and patterns within sleep epochs that may reflect specific stages or provide markers of general health conditions [11].

To address the first limitation, Explainable AI (XAI) approaches can be employed, to achieve this trust by explaining the reasoning behind accurate black-box models predictions. Broadly, these approaches fall into two categories: post-hoc and mechanistic. Post-hoc methods (e.g., LIME [12], SHAP [13]) are model-agnostic and operate only on the observed input–output relationships of a model, without examining the contribution of specific internal components, such as a specific layer of the neural network or group of layers implementing a specific functional operation. In contrast, mechanistic methods (e.g., LRP [14], CRP [15]) focus on elucidating the functional roles of the model’s layers, thereby reconstructing the sequence of transformations from input to output. Both approaches generally produce instance-level explanations, i.e. explanations related to a single input instance. Since examining the model’s decision-making across hundreds or thousands of training instances is practically unfeasible, global explanations are derived by aggregating multiple local explanations, a process that inevitably reduces the precision and fidelity of the resulting explanations relative to local ones [16].

To overcome the second limitation, a promising approach is to train the sleep-staging model to capture both sleep micro- and macrostructure. In this setup, the network would still predict the sequence of sleep stages from PSG epochs while also learning to identify and represent shared input features across epochs and subjects. These features, which influence the model’s decisions, can be interpreted as elements of sleep microstructure. Because labeled data for microstructural events are limited, the main challenge lies in enabling the network to autonomously extract the most relevant and distinctive microstructural signatures and use them to improve stage prediction. Finally, to make these representations clinically interpretable, XAI methods are needed to clarify not only the connection between PSG inputs and sleep stage outputs but also the intermediate relationships among PSG signals, sleep microstructure, and macrostructure.

In this context, a novel class of global explanations approaches, namely the prototypes explanations, are raising [17–19]. Prototypes are minimal, highly interpretable subsets of samples that serve as a distilled or condensed representation of the entire dataset [20]. Prototype explanations typically employ post-hoc or mechanistic strategies to link individual model decisions to specific prototypes, thereby providing simultaneous local and global interpretability. Prototypes can be also seen as an approximation of the model decision making in the prototype neighborhood, effectively functioning as a quantization of the input space.

A key open question in prototype-based explanations is whether the model should integrate prototypes into its decision-making or use them solely for explanation [19]. The latter approaches offer broader applicability to pre-trained models, but rely on the hypothesis of the model’s decision function continuity around prototype neighborhoods. This assumption generally holds in the final classification layer but often fails in middle layers, where the fragmented latent space causes small perturbations to produce drastically different outcomes [21, 22]. Aligning the model’s reasoning with the explanatory method is critical for trustworthyness, as misalignment can produce explanations that misrepresent the model’s actual decision-making. Conversely, constraining the architecture to rely on prototypes during prediction, requires architectural redesign and retraining and typically reduces performances due to quantization of the decision space.

**Fig. 1:**
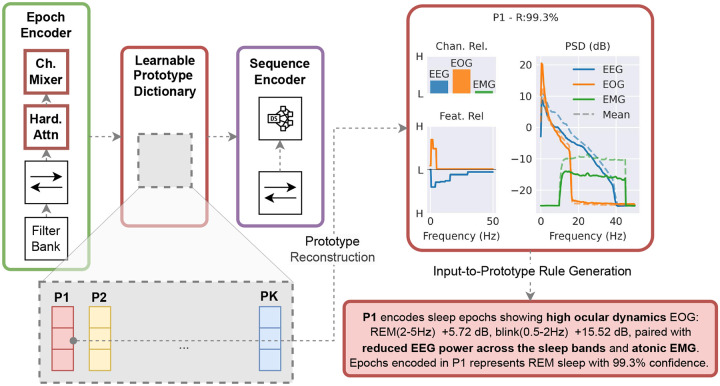
Architecture design of ProtoSleepNet (PSN). PSN extends the sequence-to-sequence architecture with an epoch encoder and a sequence encoder that incorporates a learnable dictionary, a channel mixer, and a hard attention layer. This design enables discrete and interpretable prototype assignments while maintaining model accuracy and enhancing robustness. Our framework makes it possible to reconstruct abstract prototype instances in the input space and to visualize the key information captured by each prototype, such as the power spectral densities (PSD) of the EEG, EOG, and EMG channels, together with their respective importance. In addition, a rule-generation mechanism enables these reconstructions to be translated into natural language descriptions.

In this work, we propose for the first time a prototype-based sleep staging architecture within a sequence-to-sequence framework, ProtoSleepNet (PSN), as shown in Fig 1, and demonstrate that it preserves good accuracy while providing interpretability trained on a diverse collection of datasets encompassing over 10,000 subject recordings. This interpretability is achieved through a framework-specific mechanistic explanation methodology that reveals: (1) how individual 30-second sleep epochs are matched to specific prototypes and which physiological features each prototype captures; (2) how these features can be visualized as both images, and matching rules expressed in natural language for user-oriented accessibility; and (3) how the sleep staging network processes each prototype to generate the final stage prediction.

Our system employs vector quantization to map continuous latent features onto a finite set of trainable prototype vectors stored in a codebook. During training, the model discretizes the input space by aligning continuous feature representations with their nearest codebook entries, thereby forming the prototypes. This design inherently supports the prototype explanation hypothesis, as the model’s decision function remains locally constant within each prototype’s neighborhood.

Most importantly, to demonstrate the relevance of the prototypes in a clinical context, we explore the sequence of prototypes (prototype-gram) within patients with Parkinson’s and Alzheimer’s disease. Without incorporating any information about subjects’ clinical conditions, we train a simple linear regressor on the prototype-gram to differentiate healthy subjects from patients. This way we evaluate how the autonomosly learned sleep microstructure, not only agrees with standard features outlined in the sleep scoring manuals [23], but also relate with the subject neurological condition. We further measured the information gain between sleep macro and micro structure by appling the same clinical-condition recognition pipeline to the sleep-macrostructure features.

Results demonstrate that PSN outperformed the original sequence to sequence approach, SeqSleepNet, in particular in terms of robustness to missing channels or channel occlusion attacks. In addition, the prototype-learning mechanism of PSN was quantitatively assessed by measuring the accuracy of SeqSleepNet when constrained to rely on prototypes learned post-hoc by prototype-based explanation methods.

Finally, we found that the prototype-gram effectively discriminates both Parkinson’s and Alzheimer’s disease from healthy subjects, suggesting that the encoded prototype features are not only associated with sleep patterns but also with general brain health. Further analysis reveals significant differences in prototype-gram features between clinical groups, which provides insight into how these features relate to specific diseases.

## Results

This study evaluates ProtoSleepNet (PSN) across three complementary dimensions. First, we benchmark PSN’s sleep staging performance against SeqSleepNet (SSN), the established baseline framework, establishing functional equivalence between the two architectures and PSN’s superior robustness to channel occlusion attacks. Second, we extract and analyze prototype-based explanations from PSN and systematically compare them with alternative post-hoc explainability methods applied to SSN, demonstrating superior interpretability achieved with our explainable by design method. Third, we investigate whether the learned prototypes capture clinically relevant biomarkers by assessing their discriminative capacity for identifying individuals with neurodegenerative disorders, specifically Parkinson’s disease and Alzheimer’s disease, versus healthy controls.

### PSN Benchmarks

Both PSN and SSN were trained on PSG data aggregated from 10 distinct sleep staging benchmark datasets, including over 10,000 subject recordings, with 3 channels (EEG, EOG and EMG), both in-home and lab. settings, and multiple sleep scorers. Additional details are available in the Methods section and in the supplementary.

**Fig. 2:**
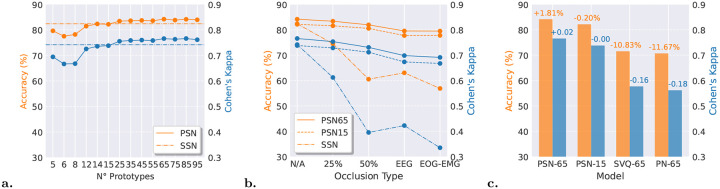
Performance evaluation of ProtoSleepNet (PSN) and SeqSleepNet (SSN) across multiple sleep staging datasets. **a.** Accuracy and Cohen’s kappa of PSN as a function of prototype count; PSN achieves performance parity with SSN using 15 prototypes and reaches peak performance with 65 prototypes. **b.** Robustness comparison between PSN (15 and 65 prototypes) and SSN under incremental channel occlusion attacks. Both PSN15 and PSN65 maintain consistent performance degradation patterns, with PSN65 exhibiting higher absolute performance reflecting increased model complexity. SSN exhibits pronounced vulnerability, with substantial metric reductions even under minimal occlusion (25% random channels). **c.** Prototype-based explainability in PSN versus post-hoc explainability methods applied to SSN: Prototypical Networks (PN) extracting cluster centroids and simVQ (SVQ) performing vector quantization on the epoch encoder latent space. Post-hoc methods incur significant performance penalties due to latent space fragmentation in SSN’s intermediate layers, highlighting the architectural advantage of intrinsic prototype learning.

We trained PSN with varying numbers of prototypes spanning from 5 to 95 to balance interpretability and performance. Fewer prototypes induce excessive quantization of the latent space, degrading model performance, whereas excessive prototypes better approximate the continuous latent distribution but compromise interpretability due to diminishing discriminability between nearby prototypes. Figure 2.a presents the mean accuracy and Cohen’s kappa across prototype counts; dataset-specific metrics are reported in the supplementary materials. PSN achieves performance parity with SSN using only 15 prototypes and reaches peak performance with 65 prototypes.

To evaluate the robustness of the PSN channel mixer layer, we implemented four distinct channel occlusion conditions: (1) a 25% probability of randomly occluding any channel within each epoch, simulating light noise spikes during the recording; (2) a 50% probability of random channel occlusion per epoch, representing medium noise spikes; (3) complete occlusion of the EEG channel for the entire recording, mimicking situations where the primary brain signal is unavailable due to major sensor failure or specific acquisition setups; (4) complete occlusion of both EOG and EMG channels for the entire recording, which simulates critical sensor failure or unavailability of the secondary modalities in the dataset.

In Figure 2.b we compare PSN across two prototype configurations: PSN with 15 prototypes (PSN15) and PSN with 65 prototypes (PSN65), against SSN under progressively channel occlusion scenarios (setups 1–4). The datasets specific results metrics are reported in tables A1, A2 and A3 of the supplementary materials. Both PSN variants demonstrate robust performance across all occlusion levels, with a consistent performance gap between PSN15 and PSN65 reflecting the latter’s increased model complexity. In contrast, SSN exhibits pronounced vulnerability to channel occlusion, achieving Cohen’s kappa ≃ 0.6 (moderate agreement) under minimal occlusion (25% random channels) and degrading further to k≃0.4 (slight-to-fair agreement) across remaining setups. PSN maintains substantially higher agreement (k>0.7) across all occlusion conditions. These findings demonstrate that PSN’s channel mixing mechanism confers robustness to channel occlusion attacks independent of prototype count, outperforming SSN.

Finally, we benchmarked PSN’s intrinsic prototype-learning mechanism against two alternative approaches for extracting prototype instances from SSN’s latent epoch encoder representation. The first approach, adapted from Prototypical Networks (PN) [24], identifies cluster centroids within the latent space. The second employs post-hoc vector quantization learning (SVQ) [25], which implements a methodology functionally equivalent to PSN’s built-in prototype learning but applied retrospectively to SSN’s learned representations. In Figure 2.c we present accuracy and Cohen’s kappa metrics for PSN and SSN when both models are constrained to rely exclusively on learned prototypes, with values on top of the bars indicating the performance delta between PSN and SSN relative to the SSN baseline. Table A4 of the supplementary material provides the datasets specific overview of the metrics. PSN’s metrics remain unchanged under this constraint, as prototype-based inference is inherent to its architecture. Performance differences between PSN and SSN are negligible when PSN uses 15 prototypes but favor PSN in the 65-prototype configuration. Conversely, constraining SSN to post-hoc prototype representations, via simVQ or PN, incurs substantial performance degradation, with accuracy approximately 10% lower and Cohen’s kappa reduced by ≃ 0.16. This disparity reflects the limited expressiveness of post-hoc prototype extraction methods, which struggle to effectively capture SSN’s decision making by discretizing the epoch encoding latent space due to its fragmentation.

### Demystifying input-to-prototype matching

After training PSN, input embeddings were extracted to explain the assignment of sleep epochs, defined as 30-second multichannel PSG windows, to specific prototypes. Because prototypes exist exclusively in embedding space, direct visualization via their components is infeasible; instead, prototypes must be reconstructed through their influence on input assignments. The reconstruction procedure relies on the principle that assignment likelihood is inversely related to latent-space distance: instances whose embeddings lie closer to a prototype have higher assignment probability.

The most straightforward approach to prototype reconstruction is to identify instances nearest to each prototype. However, this strategy has critical limitations: it assumes sufficient data density around prototypes, an assumption that may not hold for small-scale datasets, and creates strong coupling between reconstructions and the specific dataset used for prototype learning. To avoid these constraints, we leverage the prototype likelihood metric to reformulate prototype reconstruction as an optimization problem: we parameterize synthetic input-space instances and optimize them to maximize assignment probability to each target prototype, decoupling reconstructions from the original dataset. This optimization strategy can be implemented via two approaches: a fully model-driven method that initializes synthetic instances with random noise, or a hybrid strategy that seeds optimization with instances already close to the target prototype.

**Fig. 3:**
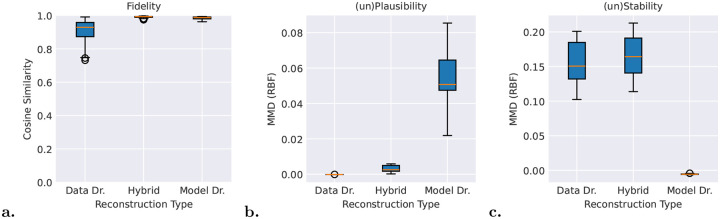
Quantitative evaluation of prototype reconstruction methods, fully data-driven, fully model-driven, and hybrid, across three complementary metrics. **a.** Fidelity: cosine similarity between reconstruction embeddings and prototype embeddings, measuring alignment with the model’s internal prototype representation. The higher scores indicate better alinement between reconstructions and prototypes. **b.** (un)Plausibility: Maximum Mean Discrepancy (MMD) distance between reconstructions and empirical instances proximate to each prototype, assessing how closely reconstructed data match the original data distribution. The lower scores indicate that the prototype reconstructions lie closer the the empirical data distribution, hence they are more similar to real empirical samples. **c.** (un)Stability: k-sample MMD distance between reconstructions across independent datasets, quantifying the dependence of reconstructions on the specific dataset used for prototype extraction. The lower scores indicate that reconstructions extracted from different datasets lie closer, hence are more similar and stable.

To quantitatively compare these three reconstruction approaches, we defined three evaluation metrics and applied them across multiple sleep staging datasets. Fidelity, displayed in Figure 3.a, measures the degree to which reconstructions align with the model’s internal prototype representation; plausibility, Figure 3.b, assesses how closely reconstructed instances match the empirical data distribution; stability, Figure 3.c, evaluates the dependence of reconstructions on the specific dataset used for prototype extraction.

Results reveal that no single reconstruction methodology dominates across all metrics. The fully data-driven method, treating nearest instances as reconstructions, exhibits lower fidelity and stability but guarantees plausibility by construction, as reconstructions are drawn directly from the empirical distribution. In contrast, fully model-driven and hybrid methods achieve comparable fidelity but diverge substantially in plausibility and stability. Fully model-driven reconstructions depend solely on initialization and are therefore dataset-independent, yielding superior stability. However, they often produce implausible synthetic instances because prototypes encode only a subset of physiological features, and non-encoded feature variations do not influence prototype distance. The hybrid method mitigates this implausibility by seeding optimization with empirically observed instances, but at the cost of reintroducing dataset dependency and reducing stability.

The fully data-driven approach is generally less preferable, as it optimizes for only one of the three metrics. The choice between hybrid and model-driven methods should be driven by application requirements. In other words, hybrid reconstruction is well-suited for clinical studies where physiological plausibility is essential, such as characterizing disease subgroups based on observed behavioral patterns, whereas model-driven reconstruction is preferable for understanding model generalization and prototype-input alignment on held-out data.

Identifying the physiological features driving prototype assignments is a crucial next step, as embedding-to-prototype distances may depend on only a subset of input characteristics, and different regions within a PSG segment can exhibit distinct physiological patterns. To address this, we developed a hierarchical decomposition strategy that relates prototype features to physiological markers across PSG channels, EEG frequency bands, EOG components (blinks, SEMs, REMs), and EMG atonia levels, and assigns each prototype a stage-specific posterior distribution. For clearer interpretation, these findings are further distilled into concise natural language rules summarizing the main physiological patterns characterizing each prototype, enabling clinicians to quickly grasp their relevance. Figure 1 illustrates both the reconstruction and the rule extracted for prototype P1 of PSN15.

Details and visualizations of local-instance-to-prototype explanations are in Appendix B and Figure B1. The methodology and global reconstructions of the PSN15 architecture are in Appendix C and Figure C2. The rule generation procedure is in Appendix D, with resulting rules summarized in Table D5.

The 15 learned prototypes by PSN15 capture distinct, stage-specific physiological patterns across sleep stages, even before incorporating sequential context. Some prototypes rely on single-channel markers (e.g., P3 and P12, dominated by delta activity), while others integrate multi-channel evidence to resolve overlapping stages. The extracted rules align closely with AASM staging criteria [23]. Wakefulness prototypes (P5, P8, P9, P11) exhibit elevated beta activity (16–30Hz), high EMG tone, and, in the case of P8, prominent REMs likely corresponding to high-frequency blinks. P5 and P11 show enhanced beta power with suppressed REMs, clearly separating wakefulness from REM sleep, whereas P8 and P9 capture alternative wake signatures through varying combinations of REM and EMG activity. The N1 prototype (P14) shows suppression of beta, gamma, and delta bands, consistent with its transitional role between wakefulness and deeper sleep. N2 prototypes (P0, P6, P7, P10, P13) display increased sigma (12–16Hz) and delta (0.5–4Hz) power along with reduced gamma and blink activity, reflecting stable spindle and slow-wave activity typical of N2. Notably, P13 shows mixed N2 and N3 specificity, illustrating the gradual delta transition between these stages. N3 prototypes (P3, P12) are dominated by elevated delta power and achieve high N3 confidence, highlighting the prevalence of slow-wave activity in deep sleep. Finally, REM prototypes (P1, P4) combine EOG activity in the 2–5Hz range with blink features (0.5–2Hz); despite overlapping frequencies, PSN distinguishes the higher-amplitude blinks from the faster, lower-amplitude REMs, successfully identifying both as complementary markers of REM physiology.

### Link between prototype-grams and neurological diseases

To measure the impact of PSN beyond sleep stage classification and assess its clinical relevance, we conducted a final experiment by leveraging the subject-specific prototype-gram, i.e., the temporal sequence of prototypes characterizing sleep dynamics across the entire night, to detect the presence of two widely studied neurodegenerative disorders: Parkinson’s disease (PD) and Alzheimer’s disease (AD).

We employed two sleep-staging datasets collected as part of a study conducted by KU Leuven, each including both healthy and patient cohorts, enabling us to compare sleep characteristics under identical recording conditions. The KU Leuven Parkinson’s dataset includes 88 participants (40 healthy controls and 48 individuals with Parkinson’s disease), and the Alzheimer Sleep Dataset includes 68 participants (32 healthy controls and 36 individuals with Alzheimer’s disease). All participants underwent full-night polysomnography with expert sleep-stage annotations. Healthy subjects were augmented considering healthy subjects coming from other 4 studies (HMC, Mass, SleepEDF and DCSM) to avoid instability of the disease detection model. A total of 800 subjects were considered in the disease recognition experiment.

We expected that more precise characterization of sleep microstructure enables extraction of information that more closely reflects an individual’s neurological status. To investigate this, prototype-grams were computed from each subject’s recording using PSN15 and PSN65, and the time spent in each prototype was used as input to distinguish healthy from pathological subjects. The same discrimination procedure was applied to an otherwise identical pipeline that instead used per-subject time-in-stage features, in order to compare sleep micro-structure (i.e., prototypes) with macro-structure (i.e., stages). A linear regressor was trained to differentiate among the three neurological conditions (healthy, Parkinson’s, and Alzheimer’s) within a Monte Carlo cross-validation framework, evaluating the AUC for each condition, as well as overall accuracy and macro-F1 score. In this framework, 8 subjects per condition were randomly selected to form the test set, with the remaining subjects used for training. This procedure was repeated 20 times to quantify the variability of the performance metrics.

**Fig. 4:**
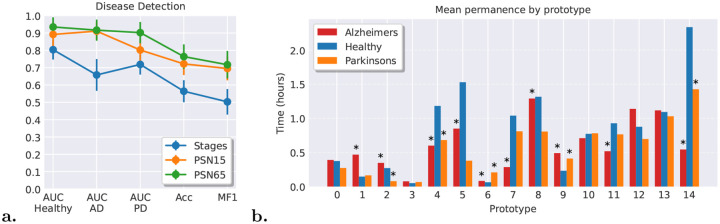
Prototype-based classification performance for detecting Alzheimer’s and Parkinson’s disease (left) subjects and analysis of the statistical differences between the 3 groups. **(a.)** Comparison of discriminatory performance between stage-based and prototype-based approaches for three neurological conditions: Healthy, Parkinson’s disease (PD), and Alzheimer’s disease (AD). The gradual increase in AUC from PSN15 to PSN65 demonstrates that detailed sleep microstructure analysis provides superior detection of neurological condition biomarkers compared to coarse, stage-level sleep macrostructure. **(b.)**Visualization of statistically significant differences (evaluated using a Mann–Whitney U test with p<0.05) in the mean time spent in each prototype of the PSN15 architecture when comparing healthy subjects with individuals diagnosed with Alzheimer’s or Parkinson’s disease. Prototypes or prototype–group bars that are absent indicate that no statistically significant difference was found relative to the healthy group.

Figure 4.a shows the performance metrics for disease detection, including AUC values per condition (Healthy, Alzheimer’s Disease, and Parkinson’s Disease), overall accuracy (Acc), and macro F1-score (MF1) across experimental configurations. PSN15 and PSN65 demonstrate progressive performance improvements compared to the stage-only baseline, revealing that finer-grained sleep microstructure characterization enhances discriminative capacity for both Alzheimer’s and Parkinson’s disease recognition. The stage-only pipeline achieves lower AUC values across all disease conditions, demonstrating that sleep macrostructure alone, whether represented by expert-labeled stage sequences, provides limited discriminative power for detecting neurological conditions. Enhanced discriminative capacity emerges exclusively when prototype count increases, revealing that finer-grained sleep microstructure encodes physiologically meaningful variation aligned with subject health status. By controlling the number of prototypes, PSN enables extraction of granular sleep-physiological information that captures individual neurological condition differences in a manner inaccessible to coarse stage-level representations.

These substantial performance differences, reflected in the marked elevation of per-condition AUC values, overall accuracy, and MF1-scores for PSN15 and PSN65 relative to the stage-only baseline, reinforce the evidence that sleep microstructure, described by characteristic prototype patterns capturing sequential stage transitions and their temporal dynamics, exhibits a more robust relationship with neurological condition than conventional sleep macro-structure (i.e., based solely on sleep stages). While sleep stage-only measures provide gross-grain descriptors of sleep architecture, they fail to capture the complexity of stage-to-stage transitional processes that distinguish healthy sleep from pathological variants. The marked performance gap underscores how disrupted sleep microstructure constitutes a more sensitive biomarker of neurodegeneration than simple alterations in stage proportions.

To characterize the physiological distinctions between groups, we performed a statistical significance analysis using the Mann–Whitney U test (p ¡ 0.05) to compare average time spent in each prototype across healthy participants and those with Alzheimer’s and Parkinson’s disease. These results are presented in Figure 4.b. We further investigated how the same prototype encodes distinct physiological features across groups by extracting per-subject reconstructions, computing associated features, and applying the same statistical test at the feature level. Multiple test correction was applied to account for multiple comparisons. A comprehensive analysis is provided in the supplementary material. Findings for prototype P4 and P14 are summarized in Table 1.

Prototypes P4 and P14 exhibit the most pronounced differences in time spent between healthy subjects and those with AD or PD. Prototype P14 represents a typical Wake-to-N2 transition state that, within the sequential sleep architecture of healthy subjects, associates with Wake, N1, and N2 stages with respective confidences of 21%, 24%, and 40%; additionally, its encoded EEG features exhibit a (weak) association with REM sleep (14% confidence). Its substantial presence throughout sleep, averaging over 2 hours, indicates well-preserved sleep structure with smooth, recurring physiological transitions between stages. In AD patients, P14 is less frequent (¡1.5 hours) and shows reduced delta-band power typical of wakefulness rather than sleep. This shifts confidence toward Wake (33%) and away from N1 and REM (16% and 5%), suggesting diffuse intermittent arousals. In PD patients, P14 occupies even less sleep time (≃0.5 hours) and aligns more with N2 and less with REM (45%, 10% confidence). The lack of increased Wake association in PD suggests that P14 reflects a disrupted architecture with direct transitions from Wake to N2, bypassing lighter sleep stages.

In healthy subjects, P4 represents a stable and well-consolidated REM state, characterized by low-amplitude, high-frequency EEG activity (similar to waking states), pronounced muscular atonia, and vigorous eye movement activity. The network assigns high confidence to the REM stage class (82%) within the sequential sleep architecture of healthy subjects, who typically spend slightly more than one hour per night in this prototype. In contrast, subjects with AD and PD show substantial contraction of P4 occupancy to approximately 30 minutes, indicating impaired REM consolidation in both neurodegenerative conditions.

In AD patients, this deficit is partially compensated by increased occupancy of the two other REM-associated prototypes (P1 and P2); however, these show lower REM confidence (P1: 74%; P2: 58%) compared to P4, indicating reduced prototype purity. This pattern suggests not a complete loss of REM sleep in AD, but rather a deterioration in the fisiological REM sleep consolidation. This interpretation is further supported by the markedly reduced REM confidence assigned to P4 within AD subjects’ sequential sleep context (64%), reflecting fragmented or intermittent REM episodes.

In contrast, PD patients do not show compensatory REM sleep in alternative prototypes. Instead, they exhibit very low occupancy of both the P4 and P2 REM-related prototypes, indicating a generalized suppression of REM sleep duration and consolidation.

A detailed examination of all statistically significant prototype differences between healthy participants and patients is provided in Appendix E of the supplementary materials, where the overall feature profiles of each prototype are reported in Tables E6 and E7.

## Discussion

In this paper, we introduce ProtoSleepNet (PSN), the first prototype-based sleep staging architecture. We demonstrate that PSN preserves and enhances both performance and robustness compared to state-of-the-art models such as SeqSleepNet [6], while explaining its decision making using prototype-explanations. We develop a novel mechanistic explanation method that reveals how input elements match to specific prototypes, which physiological features each prototype captures, how these features are visualized as images or expressed as natural language matching rules, and how the sleep staging network processes prototypes to generate stage predictions. Most importantly, we validate the clinical relevance of learned prototypes by demonstrating their ability to discriminate healthy subjects from those with Parkinson’s and Alzheimer’s disease.

The architecture we propose is a modular system that extends current state-of-the-art models following the sequence-to-sequence framework for sleep staging with high compatibility. This extension was applied to the most widely used model in the literature, SeqSleepNet, without altering its functional characteristics, in particular the structure of the epoch encoder and sequence encoder.

On the technical side, standardizing two key components of sequence-to-sequence sleep staging networks was required within PSN, i.e. temporal attention and channel mixing. Before our standardization, these components were typically implemented in the networks’ epoch encoder as (1) an additive attention layer that aggregates time-domain information within the 30-second epoch to produce a unified encoding, and (2) a concatenation of channel inputs for channel mixing. However, this approach presented two critical limitations. The soft attention mechanism in temporal attention can select non-adjacent events within the input epoch, which degrades interpretability and prevents matching to a single discrete prototype instance. Additionally, concatenating channels for mixing distributes this operation across the entire epoch encoder, making it difficult to mechanistically understand how channel information flows to the specific prototype. Furthermore, unstructured channel mixing can introduce multi-modal competition problems, where the EEG modality typically overfits during early training stages, causing the model to over-rely on this modality and become vulnerable to channel occlusion attacks. To this aim, we re-designed such operations with two novel layers: Hard Attention Masking and Channel Mixer.

The fact that ProtoSleepNet achieves accuracy comparable to or exceeding SeqSleepNet despite employing decision space quantization indicates that the selected prototypes adequately represent the network’s functional behavior. Moreover, our channel dropout mechanism effectively mitigates multi-modal competition, making the model robust to noise artifacts from temporary channel malfunctions and to complete channel absence, further demonstrating ProtoSleepNet’s applicability to resource-limited datasets.

ProtoSleepNet is an intrinsically explainable model that uses prototypes learned in the epoch encoder latent space to provide insights into its decision-making. Unlike post-hoc prototype methods, ProtoSleepNet constrains its decisions to rely on automatically learned prototypes at the epoch level. This design choice is critical for sequence-to-sequence sleep staging as identifying prototypes at the sequence encoder layer would yield prototypes spanning multiple epochs, complicating interpretability. Furthermore, the fragmented latent space in middle layers, such as the epoch encoder, prevents accurate post-hoc prototype identification that reliably describes model behavior, as demonstrated by this study evaluating the performance drop when SeqSleepNet is constrained to rely solely on post-hoc learned prototypes in the epoch encoder latent space.

Other intrinsically explainable prototype-based models include ProtoPNet [26], which learns prototypical image patches and explains by comparing input regions with these prototypes, and PIP-Net [27], which discovers prototypes self-supervisedly with spatial feature localization. While effective for images, they face key limitations in sleep staging. First, they treat prototypes as translation-invariant image objects (e.g., a paw is a paw anywhere), whereas time-frequency features are frequency-specific; a 12 Hz spindle is qualitatively different from 2 Hz delta activity. Using time-frequency patches as context-independent prototypes risks capturing physiologically meaningless, decontextualized patterns. Second, sleep staging often depends on few distinctive markers, such as spindles for N2. Learning many prototypes for such univocal features introduces redundancy and complicates interpretability. ProtoSleepNet overcomes these issues by learning abstract prototypes instead of fixed-size patches, automatically discovering variable-length temporal patterns via time-attention masking. This design captures sleep phenomena from brief events (spindles, K-complexes) to full epochs, while preserving physiological meaning and interpretability through semantically meaningful prototype representations.

Unlike approaches such as ProtoPNet that only select representative instances, our prototypical reconstruction mechanism analyzes prototypes to identify the subcomponents most influencing the input-to–prototype matching function. By formulating this as an optimization problem, the system maximizes alignment between reconstructed prototype instances and their prototypes, enabling meaningful interpretation even in low-data laboratory contexts where samples may be too sparse to reveal encoded information. We propose two complementary strategies: a fully model-driven approach and a hybrid one. Both yield reconstructions with higher fidelity to the encoded prototype; the model-driven variant supports exploratory research on general prototype properties, whereas the hybrid method balances empirical plausibility and representational fidelity by partially reintroducing data dependence. The reconstructions produced by this method closely aligned with AASM sleep scoring standards, enabling a qualitative validation of the network’s decision-making process relative to expert human scorers.

The time spent in each prototype effectively discriminated among three groups: healthy individuals, and patients with Parkinson’s or Alzheimer’s disease. Thus, it demonstrates that the prototypes capture sleep microstructure reliably reflecting health status. This approach substantially outperformed classifications based solely on sleep macrostructure or handcrafted microstructural features reported in previous studies [2]. Additionally, a statistical analysis of subject-specific, data-driven prototype reconstructions revealed significant group differences, both in the time spent within each prototype and in the distinct physiological expressions that the same prototype exhibited across subjects from different clinical groups.

On the whole, PD patients presented limited alterations evidenced by the prototypes. This can be linked to the fact that participants from the PD dataset were evaluated cross-sectionally, and had mild to moderate disease severity on the Hoehn and Yahr scale [28], ranging from II (i.e., bilateral symptoms without impairment of postural reflexes) to III (i.e., bilateral symptoms with impaired postural reflexes and consequent increased fall risk). Such limited variability within the sample did not allow to highlight possible nuances in the alterations of slow wave sleep, where higher SW density in PD has been previously associated to slower worsening of motor symptoms after 4–5 years [29]. Additionally, participants did not present signs of cognitive impairment, a symptom that has been widely associated to EEG alterations in PD, such as reductions in slow wave sleep [30].

Despite the limited variability within the PD dataset, differences with the healthy population were highlighted by the spindle-activity prototypes, with a reduction in sigma power spectral density. This finding is in agreement with previous literature reporting reduced spindle activity in PD [31], and its link to development of dementia after 4–5 years, specifically associated to reductions in spindle frequency and density and alterations in amplitude [32].

The prototypes did not highlight N1 differences between PD and healthy controls, in disagreement with meta-analytic findings reporting increased NREM1 sleep in PD [33]. However, wake-related prototypes did highlight differences between PD and healthy controls, in agreement with current evidence suggesting increased arousals [33]. The increase in wake and possible sleep fragmentation can also be linked to increased sleep disordered breathing, which is more and more identified as a contributing factor to both PD [34] and in AD [35].

Interestingly, overall cortical slowing in PD was highlighted by several prototypes. This finding is in line with a previous study by Latreille et al [36], where they further conclude that such electrophysiological changes may be associated to later development of dementia. Notably, the AD dataset consistently presented cortical slowing in the beta and gamma band, further suggesting this feature as indicative of neurodegeneration.

Finally, people with PD showed reduced REM-sleep and increased fragmentation of REM-sleep in the REM-associated prototypes. These findings are in agreement with the literature, confirming the evident reduction of REM-sleep [33]. Notably, people with PD may show other REM-sleep disturbances, such as REM-sleep without atonia, or REM-sleep behaviour disorders (RBD). Although RBD was not formally assessed in the dataset, REM-sleep without atonia may have contributed to the mis-labeling of REM sleep stages. This is an aspect that warrants caution in the selection and interpretation of the prototypes, as some Wake, N1 or N2-associated prototypes may actually contain information on REM sleep for the PD population.

The primary limitation of ProtoSleepNet is learning prototypes through supervised sleep staging, which may eliminate physiologically relevant behaviors misaligned with staging criteria or over-align prototypes to individual scorer biases. We mitigated scorer bias through training on recordings from over 10,000 subjects across multiple laboratories and geographic regions. However, a key future direction is learning prototypes in an unsupervised manner, enabling ProtoSleepNet to function as an intrinsically interpretable vocabulary for PSG signals during sleep, potentially becoming the first CLIP-like model for EEG signals, capturing sleep physiology independently of staging conventions.

Finally, we benchmarked ProtoSleepNet on sleep staging, a clinically demanding task that stands to benefit substantially from automation. This domain provides an ideal validation environment because the EEG signal exhibits highly structured, well-characterized oscillatory patterns during sleep, extensively documented in the literature and directly mappable to clinical diagnostic criteria. Our ability to align learned prototypes with established neurophysiological knowledge validates PSN’s decision-making mechanisms and demonstrates clinical fidelity. This validation positions ProtoSleepNet for deployment in other high-stakes clinical applications involving EEG analysis, such as epilepsy detection and broader neurological disorder recognition, where interpretability and performance are both critical. Beyond EEG-related use cases, PSN’s modular architecture, developed and validated on multimodal data, can be straightforwardly adapted to a wide range of time-series analysis problems, including cardiac, respiratory, and other biomedical signal processing scenarios where interpretable classification is critical.

## Methods

### Sleep Staging Datasets

A total of ten polysomnography (PSG) datasets were included in the experiments (Table 2).

Of these, four are available on the NSRR [45] (SHHS [4], MESA [38], MrOS [37] and WSC [39]); Four more datasets (MASS [41], HMC [42], SleepEDF [43] and DCSM [40]) are publicly available. The Alzheimer Sleep Dataset (ASD) [2] was sourced from a prior observational cross-sectional study on AD, eligible recordings, encompassing home-based overnight PSG, were selected from 30 healthy control (HC) subjects and 35 patients with either probable clinical AD (n = 7) or biomarker proven AD dementia (n = 28). The KU Leuven Parkinson (KPD) dataset was sourced from a retrospective study [44] on overnight PSG recordings from 48 PD and 40 HC.

All datasets share overnight PSG recordings with referenced manually annotated sleep stages. Technicians annotated sleep stages following the rules outlined by the American Academy of Sleep Medicine (AASM). Sleep stages annotations were available for each 30-seconds window of PSG. The channels used for manual sleep scoring included the EEG signal and other modalities such as electrooculography (EOG) and electromyography (EMG). These stages were divided into Wake, N1, N2, N3 (deep sleep) and REM (R).

We applied a standard state-of-the-art preprocessing pipeline to all sleep datasets [6–8], designed to isolate the frequency bands relevant for sleep analysis. Accordingly, all channels were resampled to 100Hz. EEG and EOG signals were band-pass filtered between [0.3,40]Hz, and EMG is high-pass filtered at 10Hz. If wake epochs were overrepresented compared to sleep epochs, they were reduced by cutting the initial and final part of the recording [8]. Recordings were segmented into 30-second windows of three channels. These segments were then transformed into time-frequency images by dividing each epoch into two-second windows with 50% overlap, multiplied with a Hamming window, and transformed to the frequency domain using a 256-point Fast Fourier Transform. Finally, the amplitude spectrum was power-transformed [6] and clipped to a minimum of −25dB.

The recordings were finally randomly divided into train, validation and test sets with 70%−15%−15% ratio, assuring no subject information leaking between sets, and the corresponding splits were merged to form unified training and validation sets. Each test set was evaluated separately to assess cohort-specific performance.

### A gentle introduction to the Sequence-To-Sequence Sleep Staging

Let S denote a batch of input sequences of PSG multi-channel time–frequency images. $S\in R^{L\times C\times T\times F}$, where L is the sequence length (number of consecutive 30-s epochs), C the number of channels, T the number of time frames per epoch, and F the number of frequency bins in the time–frequency representation of each epoch. According to [6] L has been set to 21, while C is equal to 3 (corresponding to EEG, EOG and EMG channels) and T and F are equal to 29, 129 respectively. We do neglect the batch dimension here for simplicity.

The sequence-to-sequence model learns a mapping $f_{SS}:S\mapsto Y,\;Y\in R^{L\times K}$, where K = 5 is the number of sleep stages and the final dimension of Y contains the predicted probabilities per stage (softmax applied across the K dimension). The architecture is commonly organized in two steps: an epoch encoder (feature extractor) and a sequence encoder. The epoch encoder, $f_{EE}$, encodes each epoch independently into a D-dimensional latent space: $H=f_{EE}\left(S\right),\;H\in R^{L\times D}$.

Operationally, $f_{EE}$ is applied on a per-epoch basis (i.e., it does not use sequence information). This way, the encoder treats each epoch independently, preventing any temporal information from leaking between epochs during epoch-level encoding.

The sequence encoder $f_{SE}$ instead models the temporal dependencies across the L adjacent latent epoch-embeddings and returns per-epoch predictions: $Y=f_{SE}\left(H\right),\;f_{SE}:R^{L\times D}\to R^{L\times K}$.

Typical choices for $f_{SE}$ include (bi-)LSTMs, GRUs, temporal convolutional networks, or Transformer encoder layers.

A common internal decomposition of the epoch encoder clarifies how intra-epoch structure is handled. One option treats each frequency bin as a sequence over time frames (length T) and projects each frame into a D-dimensional representation, possibly combining channel and frequency information within the frame: $SH=f_{\text{SEE}}\left(S\right),\;SH\in R^{L\times T\times D}$, where $f_{SEE}$ maps each time frame to a d-dim vector. An aggregation function $f_{A+}$ (e.g. additive attention) then summarizes the per-frame projections into the epoch latent: $H=f_{A+}(SH),\;H\in R^{L\times D}$. Channel aggregation can be implemented before or after $f_{SEE}$. Options include concatenation of channel embeddings followed by a projection, channel-wise attention that weights channels per frame or simple averaging or learned linear combinations across channels.

### ProtoSleepNet

ProtoSleepNet builds on the standard sequence-to-sequence sleep-staging framework by adding a small set of targeted components that improve robustness, interpretability and clinical relevance while remaining compatible with any epoch/sequence encoder pair (for example SeqSleepNet, L-SeqSleepNet, SleepTransformer). The core modules of the base sequence model (denoted here by ${(f}_{SEE})$ and $\left(f_{SE}\right)$) are kept modular and implementation-agnostic so that ProtoSleepNet can be applied on top of existing sequence-to-sequence architectures.

The principal ProtoSleepNet components are: (1) Time Hard-Attention Masking: a mechanism that selectively masks temporally local frames within an epoch’s time–frequency representation to force the model to rely on robust, non-spurious temporal cues. (2) Channel Mixing with Channel Dropout: a channel aggregation stage that learns channel combinations while optionally dropping channels during training to increase robustness to missing or noisy sensors. (3) Prototype Learning: a low-dimensional, interpretable latent layer that learns prototypical epoch patterns used for downstream classification and explanation.

These components are arranged with additive residual connections rather than as strictly chained blocks. In practice, if $\left(X_{i}\right)$ is the embedding entering the (i)-th module and $\left(g_{i}\right)$ denotes that module’s transformation, the residual update is written as $X_{i+1}=X_{i}+g_{i}\left(X_{i}\right)$. Residual coupling has two practical advantages: it (i) stabilises training by making each module learn a correction to its input, and (ii) preserves an explicit, traceable information path so the contribution of each module can be probed. To encourage each module to act as a corrective term at the start of training, module parameters are initialized so that $g_{i}(X)\approx 0$ for any inputs. This zero-ish initialization ensures the network behaves like an identity map at initialization and lets individual blocks grow meaningful corrections during training.

Explainability is further supported by computing intermediate, block-specific predictions and losses. Let $f_{FF}$ denote the feedforward projection used by the sequence encoder to map sequence embeddings to stage logits. At each residual depth i we compute ${\overset{ˆ}{Y}}_{i}=f_{FF}\left(X_{i}\right)$ and the corresponding cross-entropy loss ${ℒ}_{i}=\text{CE}\left({\overset{ˆ}{Y}}_{i},Y_{\text{true}}\right)$. Aggregation of these intermediate losses provides a diagnostic signal that measures how much each block improves classification accuracy and helps identify which module contributes most to the final prediction.

### Time-Wise Hard-Attention Masking

While the additive attention $f_{A+}$ effectively summarizes the T latent frame projections into a compact D-dimensional vector, it has two important limitations. First, its soft weights lie in [0, 1] and may assign relevance to scattered, non-adjacent time frames, which reduces temporal coherence and makes the attended pattern hard to interpret. Second, because every frame contributes proportionally to its learned weight, it is difficult to identify a small, discrete temporal region that the model actually relies on for a given epoch.

To address these issues we introduce Time-Wise Hard-Attention Masking, which forces the model to select a contiguous subsequence of frames within each epoch. The mechanism learns a binary mask M that occludes irrelevant frames and retains a compact, contiguous window of length that depends on the input. Constraining the retained frames to be adjacent produces clear, temporally coherent evidence about which parts of the epoch drive the representation.

Let the per-frame epoch projections be $SH\in R^{L\times C\times T\times D}$. We can pre-compute the set of all candidate contiguous masks with stride 1, $\bar{M}\in \{0,\;1{\}}^{M\times T}$, where M is the number of candidate windows (for example all windows of every possible length and start position).

We then score the M candidate masks from the frame projections using a learnable linear mapping $W\in R^{D\times M}$. The frame-wise mask logits are

L=SH⋅W,

and we convert these logits into per-frame probabilities over the mask set using a softmax along the mask dimension, then aggregate across frames to obtain a per-example mask vote vector

$$V=\sum_{i}^{T}\text{softmax}\left(L_{i}\right)$$

Normalising V along the mask axis yields a valid categorical distribution over the M masks for each input element. To provide an end-to-end training while producing a one-hot selection, we apply the Gumbel–Softmax relaxation: $V\mapsto \tilde{V}$ where $\tilde{V}\in [0,\;1]$ is a differentiable, approximately one-hot vector. The selected mask is assembled by multiplying the one-hot selector with the candidate masks:

$$M=\tilde{V}\cdot \bar{M}\in [0,\;1{]}^{L\times C\times T},$$

which, in the hard limit, is binary and contains a single contiguous block of ones per selection. The masked pooling that produces the epoch latent is then computed by summing only over retained frames and normalising by the number of selected frames (with a small ϵ for numerical stability):

$$H=\frac{\sum_{i=1}^{t}M_{i}⊙{SH}_{i}}{\sum_{i=1}^{t}M_{i}+\epsilon }\in R^{L\times C\times D}.$$

In practice we (1) generate $\bar{M}$ to cover a sensible range of window lengths (to limit M), (2) use a Gumbel-Softmax, and (3) apply a straight-through estimator so that evaluation uses hard masks while backpropagation benefits from the relaxed gradients.

This design yields temporally coherent, easy-to-explain epoch summaries: each epoch’s representation is explicitly derived from a single contiguous temporal segment, improving interpretability and reducing the chance that scattered, spurious frames dominate the model’s decision.

The time-wise attention masking layer encourages the network to rely on a single contiguous temporal segment within the input time-frequency representation; however, this architectural constraint does not guarantee exclusive focus on a single segment. Because the mask input SH is pre-encoded by the epoch encoder $f_{EE}$, any information leakage across temporal components within the encoder undermines the masking mechanism’s ability to enforce strict temporal localization. Consequently, we do not interpret the learned attention mask as a direct explainability tool but rather as an aggregation mechanism for combining contextual information across the sequence.

### Channel mixing and Channel Dropout

Polysomnographic (PSG) channels (EEG, EOG, EMG) provide complementary views of sleep physiology and combining these modalities typically improves staging accuracy, but naive fusion can reduce interpretability, create multimodal competition (where the model over-relies on a single, easy channel) and increase fragility to channel occlusion.

To balance performance and interpretability we introduce a configurable channel-mixing block, implemented as a residual module $f_{CM}$. Let the channel-wise encoded features be $H\in R^{L\times C\times D}$. The channel-mixed output is obtained by applying $f_{CM}$ to the channel representations and forming a residual average across channels:

$$CH=\frac{1}{C}\sum_{j}^{C}{\left(f_{CM}(H)+H\right)}_{j}$$

In our experiments $f_{CM}$ is realized as a Transformer Encoder that processes the channel vectors as a sequence and learns fusion weights; averaging across the channel axis preserves the original feature amplitude scale.

Channel mixing enables input-dependent fusion, but it can also produce multimodal competition: if one channel minimizes the training loss faster than the others, the network tends to rely on it and ignore other channels, harming robustness when that channel is noisy or missing. To counteract this, we introduce channel dropout with adaptive occlusion probability. Intuitively, channels that are individually more predictive are occluded more often during training so the model is forced to learn from the remaining modalities.

In practice, let acc_*i*_ be a recent estimate of single-channel accuracy for channel i. We set the occlusion score proportional to ${\text{acc}}_{i}$ and normalise across channels:

$$P_{\text{occl},i}=\frac{{\text{acc}}_{i}}{\sum_{j=1}^{c}{\text{acc}}_{j}},$$

so that channels with higher single-channel performance receive higher occlusion probability. During each training epoch we sample a channel permutation and occlude channels according to $P_{\text{occl}}$, forcing the network to mix and rely on multiple channels.

### Prototype Learning

Consider CM the output of the previous channels mixer layer, each element of CM is referred as ${CM}_{1},{CM}_{2}\ldots {CM}_{L}\in R^{D}$. The Prototype Learning mechanism projects each of the ${CM}_{i}$ vectors to a prototype $P_{j}\in R^{D}$ from a set of P learnable prototypes (i.e. a learnable dictionary) $LD=\left[P_{1}\ldots P_{P}\right]\in R^{P\times D}$ via a vector quantization mechanism inspired by SimVQ [25].

For each input ${CM}_{i}$, the quantized prototype $P_{j}$ is obtained by selecting its most similar element in LD:

$$j^{*}=\text{arg}\underset{j\in \{1,\ldots ,P\}}{\text{max}}sim\left({CM}_{i},P_{j}\right),\;sim\left({CM}_{i},P_{j}\right)=\frac{{CM}_{i}^{⊤}P_{j}}{\left\|{CM}_{i}\right\|\left\|P_{j}\right\|}$$

The output of the Prototype Layer is thus the matrix of quantized prototypes corresponding to each input encoding:

$$P=\left[P_{1},P_{2}\ldots P_{L}\right]\in R^{L\times D}$$

Since the discrete assignment is non-differentiable, the Prototype Layer uses a straight-through estimator to allow gradient flow. The quantised output P is passed forward, but the gradient is copied from the prototype to the input encoding:

P=CM+sg(P-CM)

where sg(·) denotes the stop-gradient operator.

This operation ensures that the forward pass uses the discrete prototype $P_{j}$, while the backwards pass updates the encoder to produce encodings closer to the prototype entries. Additionally, a commitment loss is employed to encourage the encodings to commit to their assigned prototypes:

$$L_{\text{commit}}=2-sim(CM,P)$$

### Demistifying Input-to Prototype Matching

Let’s consider the function flow before the prototype layer applied to a single epoch instance $\bar{S}$ of dimension C×T×F, and let’s name it the ProtoSleepNet epoch encoder $f_{EE}$:

$$\bar{CM}=f_{EE}(\bar{S})\in R^{D}$$

We can note that the prototype layer $f_{PL}$ is applied to the output of the epoch encoder, i.e. $P=f_{PL}(\bar{CM})\in R^{D}$. Let’s consider the problem to understand how the input $\bar{S}$ is related to one of these prototypes, say $\bar{P}$.

The likelihood of the input $\bar{S}$ to be assigned to the prototype $\bar{P}$ is given by the similarity between the two, with an higher value indicating a stronger match, suggesting that the input $\bar{S}$ is more likely to be associated with the prototype $\bar{P}$. If we want to estimate the relevance of the input $\overset{‾}{S}$ to the prototype $\bar{P}$, we can backpropagate $sim(\cdot ,\bar{P})$ towards the i-input feature component:

$$ℛ\left({\bar{S}}_{i}\right)=\frac{\partial \text{sim}(\bar{S})}{\partial {\bar{S}}_{i}}$$

Note that $ℛ\left({\bar{S}}_{i}\right)$ is the relevance of the i-th element of the tensor $\overset{‾}{S}$. We can define $ℛ(\bar{S})$ of the same dimension as the input $\bar{S}\in R^{C\times T\times F}$, and it quantifies the relevance of each input element to the prototype $\bar{P}$. A higher value in $ℛ\left({\bar{S}}_{i}\right)$ indicates that the corresponding input element contributes more significantly to the match with the prototype. Usually we are more interested in understanding how the similarity changes using a reference point, i.e., the center of the distribution $S^{\emptyset }$, and we can compute this value by integrating the relevance [?]:

$${ℛ}^{*}\left({\bar{S}}_{i}\right)=\left|{\bar{S}}_{i}-S_{i}^{\emptyset }\right|\cdot \int_{0}^{1}\frac{\partial \text{sim}\left(S^{\emptyset }+\alpha \cdot \left(\bar{S}-S^{\emptyset }\right)\right.}{\partial {\bar{S}}_{i}}d\alpha$$

where the integration is performed along the straight path from $S^{\emptyset }$ to $\bar{S}$ in the input space $R^{C\times T\times F}$.

Note that $ℛ*\left({\bar{S}}_{i}\right)$ satisfies completeness [46] thanks to the fundamental theorem of calculus:

$$\sum_{i}^{C\times T\times F}{ℛ}^{*}\left({\bar{S}}_{i}\right)=sim(S)-sim\left(S^{\emptyset }\right)$$

The supplementary material provides an illustrative example of local instance-to-prototype-matching explanation.

### From Local to Global Perspective

The local perspective provides insights into how a specific input epoch relates to a prototype. However, when analyzing large datasets with thousands of sleep epochs, this approach can become inefficient and cumbersome. To address this limitation, we introduce a *global perspective* that explains general matching rules between input instances and prototypes.

The first problem is to assign interpretable semantic structure to a prototype $\bar{P}$ by constructing a reconstruction $R^{1}\in R^{C\times T\times F}$. A straightforward fully data driven approach is to aggregate input instances from the training dataset that maximize similarity to the prototype. However, limited training data may yield insufficient exemplar density around the prototype to characterize its neighborhood adequately. To address this limitation, we define a fidelity metric as the similarity between the reconstruction’s latent projection and the prototype, quantifying how well the reconstruction captures the prototype’s internal representation.


$$\text{Fidelity}(R)=sim\left(f_{EE}(R),\bar{P}\right)$$

Conversely, when multiple data distributions are available, a secondary problem arises: determining how reconstructions derived from different distributions diverge. To quantify this phenomenon, we compute Maximum Mean Discrepancy (MDM) between distributions as a stability metric, measuring the consistency of reconstructions across independent datasets.


$$\text{Stability}\left(R^{1},R^{2}\ldots \right)=MDM\left(R^{1},R^{2}\ldots \right)$$

In order to tackle the pour fidelity problem that may arise if a non sufficient number of instances is found in the prototype neighborhood we can perform a gradient-based optimization of the prototypes by maximising the reconstruction fidelity, i.e. we can train a set of parameters $\bar{R}$ to maximise the similarity. An in important step in this direction is to choose the initialization parameters of $\bar{R}$, this way we can define a fully model-driven approach that initialize those parameters with random units, and an hybrid approach that relies on using the closer instances drawn from the distribution as initializations. On the other hand producing synthetic instances rises the problem of the plausibility of the reconstruction, i.e. how far they are from the actual data distribution. This behaviour can be evaluated by computing again the MDM between the original reconstruction R and the optimized one $\bar{R}$.

To mitigate poor fidelity arising from insufficient density around prototypes, we employ gradient-based optimization to maximize reconstruction fidelity. Specifically, we train a parameter set $\bar{R}$ to maximize similarity to the prototype. A critical design choice is the initialization strategy for $\bar{R}$: a fully model-driven approach initializes parameters with random noise, while a hybrid approach seeds optimization with instances from the original distribution proximate to the prototype. However, synthetic reconstruction introduces a secondary problem: plausibility, i.e., the degree to which optimized reconstructions remain distributed near the empirical data distribution. To evaluate plausibility, we compute distance correlation between the original reconstruction R and the optimized reconstruction $\bar{R}$, quantifying divergence from the natural data manifold.


$$\text{Plausibility}(\bar{R})=MDM(\bar{R},R)$$

Supposed now to have a batch (256 in our case) of reconstructions for each prototype $\bar{P}$, what is left apart is to understand how the specific components of a reconstruction impact the similarity with the prototype. A straightforward way to understand this would be to compute the relevance of each reconstruction with respect to the center of the distribution as discussed in the local perspective. This approach on the other hand, requires to understand the sufficient amount of integration points for each batch-instance and provides a relevance map in the same space of the input reconstruction, which is tipically too-detailed and overcomplicated to interpret. Instead we are interested in understanding how specific features of the input-reconstruction space affect the similarity, in particular for the EEG channel we are interested in the role of each specific brain wave for the EOG channel we are interested in the role of both slow and rapid eye movents and blinks, and for the EMG channel we are interested in the general role of muscle tonicity. Thanks to this observation and to the completeness property of the local relevance, we can approximate the relevance of each feature with an occlusion strategy computing the difference in the similarity before and after the occlusion of the feature with the respective distribution center values.

Given a batch of reconstructions (256 in our experiments) for each prototype $\bar{P}$, the remaining challenge is to determine which specific reconstruction components most strongly influence similarity to the prototype. A direct approach would compute element-wise relevance via local explanation methods (as discussed previously); however, this requires a sufficient amount of integration points for each instance, and yields a relevance map in the original input space, which is typically too granular and complex for clinical interpretation. Instead, we seek feature-level interpretability targeting physiologically meaningful components: for EEG signals, the contribution of specific frequency bands (delta, sigma, etc.); for EOG signals, the roles of slow eye movements, rapid eye movements, and blinks; and for EMG signals, the overall contribution of muscle tonicity. Leveraging the completeness property of local relevance, we approximate feature-level importance through an occlusion strategy: we compute the similarity change induced by replacing each feature with its distribution center value, thereby quantifying the feature’s contribution to prototype matching.

Finally, per-instance explanations are aggregated across the batch dimension for each prototype. Each feature’s relevance is converted into a natural-language rule comprising three components: (1) the feature’s relevance value, (2) the mean power of that feature in the prototype reconstruction, and (3) a directional indicator (↑ or ↓) specifying whether the reconstruction value exceeds or falls below the expected baseline. A relevance threshold (0.05 in our experiments) filters rule statements, retaining only features with sufficient influence on prototype-reconstruction matching.

## Supplementary Material

This is a list of supplementary files associated with this preprint. Click to download.

- ProtoSleepNet5.pdf

## Figures and Tables

**Table 1:** Feature profiles and stage confidence distributions for prototype P4 and P14 in healthy controls and patients with Alzheimer’s and Parkinson’s disease. EEG spectral bands, from low frequency δ to high frequency γ, EOG activity, i.e. slow-eye movements (SEMs) and rapid-eye movements (REMs), and EMG atonia are expressed in dB; stage confidences (from Wake to REM) indicate posterior probabilities within sequential sleep context.

P.ID	Feat	Healthy	Alzheimers	Parkinsons	P.ID	Healthy	Alzheimers	Parkinsons
4	δ	6.39	1.07*	5.50	14	6.79	2.70*	6.72
	θ	2.25	−2.97*	2.15		3.42	−0.81*	4.05
	α	−1.01	−6.07*	−0.59		0.46	−4.47*	1.33
	σ	−4.55	−8.55*	−4.01		−2.43	−6.74*	−2.48
	β	−8.21	−11.04*	−8.20		−7.30	−10.29*	−7.82
	γ	−14.95	−14.98	−16.19		−13.86	−14.87	−15.76*
	SEMs	14.33	12.50	11.69		9.33	6.28*	4.18*
	blinks	13.95	14.58	12.84		9.95	9.2649	6.45*
	REMs	6.83	6.56	4.93*		4.39	3.14	1.12*
	(>5Hz)	−7.54	−9.81*	−10.66*		−6.93	−9.65*	−10.94*
	EMG	−17.40	−14.07*	−15.48		−11.69	−8.92	−10.58
	Wake	3%	9%*	3.2%		21%	33%*	21.8%
	N1	6%	7.3%	4.5%		24%	16%*	21.9%
	N2	8%	11.6%	10.62%		40%	41.4%	45%*
	N3	0%	2%*	2.8%		1%	4%*	1.4%
	REM	82%	64%*	76.5%		14%	5%*	10%*

Significance markers: *p<0.05 (Mann–Whitney U test with multiple comparison correction).

**Table 2:** Summary of EEG datasets used in this study, including the number of recordings, demographic characteristics, total number of annotated sleep epochs, and dataset origin.

Dataset	# of subjects	Age (median, Q1-Q3)	Sex (%F)	Sleep Epochs	Origin
SHHS [4]	5,793	63.0 (55.0, 72.0)	52.36%	5,782,715	USA
MrOS [37]	2,898	76.0 (72.0, 80.0)	0.00%	3,624,129	USA
MESA [38]	2,055	68.0 (62.0, 76.0)	53.58%	2,448,749	USA
WSC [39]	1,113	50.5 (56.4, 62.0)	46%	1,015,564	USA
DCSM [40]	255	NA	NA	578,939	Denmark
MASS [41]	200	38.3 (std: 18.9)	51.50%	228,870	Canada
HMC [42]	151	53.9 (std: 15.4)	43.6%	137,243	Netherlands
SleepEDF [43]	78	57.0 (38.0, 72.75)	52.56%	196,350	Netherlands
KPD [44]	88	66.5 (62.0, 72.0) HC - 67.5 (60.0, 70.3) PD	40% HC - 37.5% PD	90,086	Belgium
ASD [2]	68	70.63 (std: 7.1) HC - 74.63 (std: 6.5) AD	63.3% HC - 45.7% AD	69,809	Belgium

## Data Availability

A total of 10 polysomnography (PSG) datasets were included in the experiments. 4 out of six are available on the NSRR [45] archive (SHHS [4], MESA [38], MrOS [37], WSC [39]); 2 are available on the PhysioNet (SleepEDF [4] and HMC [4]); 2 of the remaining datasets (MASS [41] and DCSM [40]) are publicly available (under request) and the final 2 datasets have been gathered by the KU Leuven. This data is under restricted access and can be made available upon reasonable request to author MG, pending ethical approval and a data transfer agreement..

## Code Availability

The PyTorch implementations of both ProtoSleepNet and SeqSleepNet architectures are available as a part of the PhysioEx library [47], together with the data loaders, splits, and seeds used in this work. The experimental setups for models training, prototypes reconstruction, explanations and disease detection are available via GitHub
